# Current Trends of Cardiovascular Risk Determinants in Pakistan

**DOI:** 10.7759/cureus.3409

**Published:** 2018-10-04

**Authors:** Afrose Liaquat, Qamar Javed

**Affiliations:** 1 Biochemistry, Shifa College of Medicine, Islamabad, PAK; 2 Biochemistry, Quaid-I-Azam University, Islamabad, PAK

**Keywords:** cardiovascular disease, risk factors, dyslipidemia, coronary artery disease

## Abstract

We carried out a case control study to determine the prevalence of various cardiovascular risk factors in a Pakistani population. A total of 835 patients (555 males and 280 females) and 794 control subjects (486 males and 308 females) were recruited in this study. Patients with documented history of coronary artery disease (CAD) were included. We assessed major risk factors such as age, body mass index (BMI), hypertension and dyslipidemia, using pre-specified definitions. A comparative analysis of the biochemical and clinical parameters was carried out between controls and patients using student’s t test. We observed that the cardiovascular disease (CVD) risk factors were more prominent in the patient group as compared to the controls (P < 0.05). In the whole studied population females had increased levels of total cholesterol (TC) (P = 0.01), triglyceride (TG) (P = 0.02), and very low density lipoprotein cholesterol (vLDL-C) (P = 0.02) as compared to males. Among patients group all the risk factors were significantly higher and more prevalent in females when compared with male patients (P < 0.05). The study population was also analyzed according to the smoking status and BMI to study the effect of these risk factors independently. The smokers and study subjects with raised BMI had significantly raised blood pressure and cholesterol levels. The role of age as a risk factor was also investigated in the current study. The persons with age ≤45 years had the highest levels of lipid profile including TC, TG, low density lipoprotein cholesterol (LDL-C), vLDL-C and high density lipoprotein cholesterol (HDL-C) among the three (≤45, 46–55, ≥56 years) groups (P < 0.05). In conclusion, the present study demonstrates an increased propensity of CVD risk factors at a younger age with female preponderance. Moreover, hypertension and dyslipidemia are the most prominent of the risk factors.

## Introduction

The cardiovascular disease (CVD) is consistently budding as the most prevalent cause of mortality worldwide. The epidemiological transition in the 20th century has placed CVD as the principal cause of global disability. According to the global health projections, it is going to remain the foremost cause of mortality in 2030 [1]. CVD and its atherothrombotic complications develop as a consequence of life style, environmental factors and genetic susceptibility.

Risk factors for CVD have been characterized as conventional or classical and novel [2]. The role of these risk factors is still primary in socio-deprived countries like Pakistan. The influence of an individual risk factor on the CVD varies greatly but the presence of numerous risk factors synergistically works and ultimately affects the CVD outcome. Most of the risk assessment methods used currently have been derived from studies on the developed countries, however, in low and middle income populations no algorithm has been developed yet [2]. Risk estimation score based on the Framingham study is the most commonly used method but it has led to the over or under estimation of the risk factors [3]. Zhu et al. have developed a synthetic predictor from 16 biomarkers that will provide assistance in the CVD risk prediction by simply using the data from routine health check-up [4]. The risk scoring method for the South Asians must include all the specific variables involving the accurate estimation of the risk factors in South Asia. Given the complex population structure of the region these include factors involving population dynamics, cultural diversity, comorbidities, socioeconomic profiles, linguistics and lifestyle.

The Asian region has been documented to have a higher CVD burden as compared to the western populations, and majority of this burden is held by the economically disadvantaged populations that are mainly in the South Asian region [5]. Pakistan is among these under-developed regions equally affected by the CVD epidemic as the rest of the world, however, there is very limited documented data regarding this problem. The current study aims not only to contribute to the scientific understanding of the disease but also help in the development of appropriate strategies regionally to prevent CVD.

## Materials and methods

Study population

We carried out a case-control study to determine the prevalence of various cardiovascular risk factors in a Pakistani population. The data included here is from the city of Islamabad, Pakistan. The patient samples were collected from cardiology outpatient department (OPD) of Shifa International Hospital, and Pakistan Institute of Medical Sciences, Islamabad, whereas control samples were collected from the general OPD who were attending for routine health checkup, the medical students, and volunteers from the general population. The whole study population belonged to middle socio-economic group. A total of 835 patients (555 males and 280 females) and 794 control subjects (486 males and 308 females) were included in this study. The work presented here was approved by Institutional Review Board (IRB) Shifa International Hospital, Islamabad. Informed written consent was obtained from the study participants according to the Declaration of Helsinki.

Inclusion criteria for the study

A questionnaire was designed to characterize both patients and control subjects. Questionnaire included personal information (name, sex, age, etc.); risk factor information (smoking, etc.); record of physical measures (weight, height, blood pressure, etc.) and medical history (disease profile and clinical symptoms). Patients with documented history of coronary artery disease (CAD) were included. Diagnostic tests for CVD included electrocardiography (ECG), chest X-ray, echocardiography and angiography.

The controls were unrelated healthy individuals representing the same geographic location and ethnic population as the patients. Their clinical histories were reviewed by two independent cardiologists who were unaware of the objectives of study. Control subjects had no history of cardiac diseases, no symptoms of other atherosclerotic vascular diseases and had normal electrocardiogram. All the control subjects were sampled to match the CVD patients for age.

Metabolic profiles

Blood samples were collected from study participants for the determination of total cholesterol (TC), triglycerides (TG), and high-density lipoprotein cholesterol (HDL-C). These blood samples were transferred into sterile plain serum tubes and allowed to clot at room temperature for one hour. The tubes were then centrifuged (Centrifuge 5417R, eppendorf) at 4000 rpm for five minutes to separate serum from cellular components and kept at -80°C for further analysis. Estimation of TC, TG, and HDL-C was carried out in our laboratory using AMP Diagnostics kits (Austria). Low-density lipoprotein cholesterol (LDL-C) and very low density lipoprotein cholesterol (vLDL-C) were then calculated by using the standard formula: vLDL-C = TG/5 and LDL-C = TC + (HDL-C + vLDL-C). All these metabolic profiles were estimated in mg/dL unit.

Definitions

According to the revised criteria for Asian population the category of overweight was defined as body mass index (BMI) ≥ 25 kg/m^2^ [6]. Blood pressure (BP) was recorded using a standard mercury sphygmomanometer. At least two readings at 10 minute interval were recorded and in case of an abnormal reading, another reading was obtained after 30 minutes. Systolic blood pressure (SBP) of 140 mm Hg or greater and diastolic blood pressure (DBP) of 90 mm Hg or greater was diagnosed as hypertension (HTN) [7].

Statistical analysis

All the statistical analysis was performed using IBM SPSS Version 22.00 (Armonk, NY). Clinical and biochemical variables were expressed as mean ± SD and evaluated by student’s t test. One way analysis of variance (ANOVA) followed by post hoc Tukey’s test was applied to compare the age groups in the study population. P value < 0.05 was considered statistically significant.

## Results

Baseline, clinical and biochemical characteristics of the study population

The baseline, clinical and biochemical features are summarized in Table 1. Eight hundred and thirty-five patients with mean age of 53.19 ± 11.85 and 794 healthy controls with mean age of 53.76 ± 9.7 were analyzed in this case-control study. The patient’s group had significantly higher BMI, SBP, DBP and lipid profile levels as compared to controls (P < 0.05).

**Table 1:** Clinical and biochemical characteristics of control subjects and patients with cardiovascular disease. Values are given as means ± SD. ^a ^*P* value calculated by Student’s independent samples *t* test (unpaired). ^* ^*P* value (<0.05) indicates statistical significance. ^* ^*P* values were calculated on log-transformed scale. n: Number of subjects; BMI: Body mass index; BP: Blood pressure; TG: Triglycerides; HDL-C: High-density lipoprotein cholesterol; LDL-C: Low-density lipoprotein cholesterol; SD: Standard deviation; TC: Total cholesterol; vLDL-C: Very low density lipoprotein cholesterol.

Characteristics	Controls (n = 794)	Patients (n = 835)	* *P value ^a^
Age (years)	53.76 ± 9.7	53.19 ± 11.85	0.291
BMI (Kg/m^2^)	24.82 ± 3.62	25.24 ± 3.60	0.01^*^
Systolic BP (mm Hg)	119.55 ± 11.01	136.91 ± 24.63	<0.0001^*^
Diastolic BP (mm Hg)	82.44 ± 6.98	86.68 ± 13.65	<0.0001^*^
TC (mg/dL)	152.67 ± 25.11	168.43 ± 49.98	<0.0001^*^
TG (mg/dL)	137.04 ± 58.31	147.34 ± 67.80	0.001^*^
LDL-C (mg/dL)	85.69 ± 24.50	98.98 ± 37.48	<0.0001^*^
HDL-C (mg/dL)	40.03 ± 12.68	35.50 ± 11.54	<0.0001^*^
vLDL-C (mg/dL)	29.95 ± 10.69	29.5 ± 13.57	<0.0001^*^
TC: HDL-C	4.07 ± 1.11	5.04 ± 1.85	<0.0001^*^

Gender-based analyses of the study population

The study population was divided on the basis of gender to analyze the clinical and biochemical characteristics between males and females (Table 2). The study population consisted of 64% (n = 1041) males with mean age of 53.38 ± 11.15 and 36% (n = 588) females with mean age of 53.61 ± 10.33 years. The BMI was significantly more in the males whereas, other risk factors like TC, TG and vLDL-C were significantly increased in female group. Blood pressure, LDL-C, HDL-C and TC: HDL-C showed insignificant differences between the study groups.

**Table 2:** Clinical and biochemical characteristics of male and female subjects in the overall study population. Values are given as means ± SD. ^a ^*P* value calculated by Student’s independent samples *t* test (unpaired). ^* ^*P* value (<0.05) indicates statistical significance. ^* ^*P* values were calculated on log-transformed scale. n: Number of subjects; BMI: Body mass index; BP: Blood pressure; TG: Triglycerides; HDL-C: High-density lipoprotein cholesterol; LDL-C: Low-density lipoprotein cholesterol; SD: Standard deviation; TC: Total cholesterol; vLDL-C: Very low density lipoprotein cholesterol.

Characteristics	Male (n = 1041)	Female (n = 588)	P value ^a^
Age (years)	53.38 ± 11.15	53.61 ± 10.33	0.68
BMI (Kg/m^2^)	25.17 ± 3.49	24.79 ± 3.82	0.04^*^
Systolic BP (mm Hg)	128.48 ± 20.46	128.40 ± 22.20	0.93
Diastolic BP (mm Hg)	84.62 ± 10.79	84.60 ± 11.70	0.96
TC (mg/dL)	160.26 ± 40.37	165.20 ± 39.72	0.01^*^
TG (mg/dL)	141.21 ± 65.77	148.72 ± 60.31	0.02^*^
LDL-C (mg/dL)	92.42 ± 31.35	92.63 ± 34.48	0.9
HDL-C (mg/dL)	37.33 ± 12.62	38.39 ± 11.75	0.09
vLDL-C (mg/dL)	28.24 ± 13.15	29.74 ± 12.06	0.02^*^
TC: HDL-C	4.60 ± 1.59	4.5 ± 1.64	0.41

To further inspect the risk factors, the patient group was divided on the basis of gender and then analyzed (Table 3). The female patients (n = 280) had significantly higher SBP, TC, TG, LDL-C and vLDL-C when compared with male patients (n = 555). The DBP and HDL-C did not show any significant differences among both groups.

**Table 3:** Clinical and biochemical characteristics of female and male subjects in the patient group. Values are given as means ± SD. ^a ^*P* value calculated by Student’s independent samples *t* test (unpaired). ^* ^*P* value (<0.05) indicates statistical significance. ^* ^*P* values were calculated on log-transformed scale. n: Number of subjects; BMI: Body mass index; BP: Blood pressure; TG: Triglycerides; HDL-C: High-density lipoprotein cholesterol; LDL-C: Low-density lipoprotein cholesterol; SD: Standard deviation; TC: Total cholesterol; vLDL-C: Very low density lipoprotein cholesterol.

Characteristics	Male (n = 555)	Female (n = 280)	* *P value ^a^
Age (years)	53.25 ± 11.86	53.06 ± 11.84	0.83
BMI (Kg/m^2^)	25.18 ± 3.54	25.37 ± 3.72	0.45
Systolic BP (mm Hg)	135.39 ± 23.47	139.92 ± 26.56	0.01^*^
Diastolic BP (mm Hg)	86.21 ± 13.35	87.61 ± 14.22	0.16
TC (mg/dL)	164.16 ± 49.62	176.92 ± 49.70	<0.0001^*^
TG (mg/dL)	141.67 ± 68.55	158.58 ± 64.93	0.001^*^
LDL-C (mg/dL)	95.78 ± 36.24	105.31 ± 39.13	0.001^*^
HDL-C (mg/dL)	35.17 ± 11.81	36.16 ± 10.99	0.24
vLDL-C (mg/dL)	28.33 ± 13.70	31.81 ± 13.02	<0.0001^*^
TC: HDL-C	4.97 ± 1.86	5.19 ± 1.82	0.1

Characteristics of the study population according to the smoking status and BMI

The division of the study population according to the smoking status and BMI (≤25 and ≥25 kg/m^2^) was done to study the effect of these risk factors independently. The smokers (n = 447) had significantly raised blood pressure and low HDL-C and TC: HDL-C when compared with non-smokers (n = 1179). The other risk factors (TC, TG, LDL-C, and vLDL-C) were also high in the smokers group but the results were insignificant (Table 4).

**Table 4:** Clinical and biochemical characteristics of smokers and non-smokers in the study population. Values are given as means ± SD. ^a ^*P* value calculated by Student’s independent samples *t* test (unpaired). ^* ^*P* value (<0.05) indicates statistical significance. ^* ^*P* values were calculated on log-transformed scale. n: Number of subjects; BMI: Body mass index; BP: Blood pressure; TG: Triglycerides; HDL-C: High-density lipoprotein cholesterol; LDL-C: Low-density lipoprotein cholesterol; SD: Standard deviation; TC: Total cholesterol; vLDL-C: Very low density lipoprotein cholesterol.

Characteristics	Smokers (n = 447)	Non-smokers (n = 1179)	* *P value ^a^
Age (years)	52.74 ± 10.94	53.76 ± 10.82	0.09
BMI (Kg/m^2^)	25.23 ± 3.46	24.95 ± 3.66	0.16
Systolic BP (mm Hg)	131.80 ± 21.97	127.18 ± 20.65	<0.0001^*^
Diastolic BP (mm Hg)	85.38 ± 11.56	84.30 ± 10.94	0.08
TC (mg/dL)	164.22 ± 43.50	161.18 ± 38.88	0.17
TG (mg/dL)	146.59 ± 72.44	142.96 ± 60.47	0.3
LDL-C (mg/dL)	93.31 ± 32.15	92.15 ± 33.02	0.52
HDL-C (mg/dL)	36.19 ± 10.89	38.28 ± 12.79	0.002^*^
vLDL-C (mg/dL)	29.31 ± 14.48	28.59 ± 12.09	0.3
TC: HDL-C	4.81 ± 1.74	4.48 ± 1.55	<0.0001^*^

The study subjects with BMI ≥25 kg/m^2^ (n = 765) also had significantly elevated SBP, DBP, TC, LDL-C and TC: HDL-C (Table 5) when compared to individuals with BMI ≤25 kg/m^2^ (n = 864).

**Table 5:** Clinical and biochemical characteristics of the study population with respect to body mass index. Values are given as means ± SD. ^a ^*P* value calculated by Student’s independent samples *t* test (unpaired). ^* ^*P* value (<0.05) indicates statistical significance. ^* ^*P* values were calculated on log-transformed scale. n: Number of subjects; BMI: Body mass index; BP: Blood pressure; TG: Triglycerides; HDL-C: High-density lipoprotein cholesterol; LDL-C: Low-density lipoprotein cholesterol; SD: Standard deviation; TC: Total cholesterol; vLDL-C: Very low density lipoprotein cholesterol.

Characteristics	BMI ≤ 25 (n = 864)	BMI ≥ 25 (n = 765)	* *P value ^a^
Age (years)	53.25 ± 11.27	53.70 ± 10.38	0.4
Systolic BP (mm Hg)	126.62 ± 19.93	130.52 ± 22.17	<0.0001^*^
Diastolic BP (mm Hg)	83.60 ± 11.32	85.76 ± 10.79	<0.0001^*^
TC (mg/dL)	159.04 ± 39.87	165.43 ± 40.31	0.001^*^
TG (mg/dL)	143.70 ± 62.74	144.17 ± 65.31	0.88
LDL-C (mg/dL)	89.92 ± 32.89	95.41 ± 31.82	0.001^*^
HDL-C (mg/dL)	37.25 ± 11.11	38.23 ± 13.54	0.112
vLDL-C (mg/dL)	29.74 ± 12.54	28.83 ± 13.06	0.18
TC: HDL-C	4.52 ± 1.64	4.63 ± 1.57	<0.0001^*^

Clinical and biochemical characteristics of the study population according to age

The role of age as a risk factor was also investigated in the current study and the samples were segregated according to their respective age (Table 6). The persons with age ≤45 years (n = 398) had the highest levels of lipid profile including TC, TG, LDL-C, vLDL-C and HDL-C among the three (≤45, 46-55, ≥56 years) groups (P < 0.05). The comparison of the risk factors within groups revealed no significant difference between the age groups ≤45 and 46-55 years (n = 584). However, the group with age ≥56 years (n = 647) showed significantly low levels of lipid profile (TC, TG, LDL-C, HDL-C, vLDL-C) when compared with other groups independently.

**Table 6:** Clinical and biochemical characteristics of the study population according to age. Values are given as means ± SD; *P* value calculated by one way analysis of variance (ANOVA). Tukey’s post hoc test applied for the comparison between groups; *P* value (<0.05) indicates statistical significance. ^a^: ≤45 vs 46-55, ≥56; ^b^: 46-55 vs ≥56; * <0.05; ** <0.01; *** <0.0001. n: Number of subjects; BMI: Body mass index; BP: Blood pressure; TG: Triglycerides;  HDL-C: High-density lipoprotein cholesterol; LDL-C: Low-density lipoprotein cholesterol; SD: Standard deviation; TC: Total cholesterol; v-LDL: Very low density lipoprotein cholesterol.

Characteristics	Age ≤45 yrs (n = 398)	Age 46-55 yrs (n = 584)	Age ≥56 yrs (n = 647)	P value
BMI (Kg/m^2^)	24.92 ± 3.74	24.91 ± 3.54	25.22 ± 3.60	0.24
Systolic BP (mm Hg)	128.51 ± 19.87	128.61 ± 21.03	128.27 ± 21.90	0.95
Diastolic BP (mm Hg)	85.36 ± 11.24	84.21 ± 11.26	84.52 ± 10.92	0.27
TC (mg/dL)	169.65 ± 43.77	164.26 ± 39.37	155.36 ± 37.55^a,b***^	<0.0001
TG (mg/dL)	150.54 ± 64.65	143.62 ± 62.71	140.12 ± 64.37^a*^	0.03
LDL-C (mg/dL)	98.07 ± 38.80	93.60 ± 30.50	88.08 ± 29.28^ a***b**^	<0.0001
HDL-C (mg/dL)	38.65 ± 13.07	38.41 ± 12.59	36.50 ± 11.49^ a,b*^	0.005
vLDL-C (mg/dL)	30.10 ± 12.93	28.72 ± 12.54	28.02 ± 12.87^ a*^	0.03
TC: HDL-C	4.68 ± 1.62	4.57 ± 1.72	4.51 ± 1.49	0.24

## Discussion

The present study aims to investigate the cardiovascular risk factors among a Pakistani population. The results demonstrated a high prevalence of multiple cardiovascular risk factors, including deranged lipid profile, elevated blood pressure, smoking, and increased BMI in the study population. Hussain et al. reviewed the assessment of cardiovascular risk in the South Asian population, and documented the increased prevalence of risk factors (traditional and unconventional) in these populations [2]. Moreover, it was also documented that the risk profiles of these populations are not only different between countries but diversity also exists within one population [2]. In a recent review conducted by Barolia and Sayani major modifiable risk factors in Pakistani population were identified [8]. Lack of population data for the cardiovascular risk factors was highlighted as only two population studies have been conducted to estimate the actual prevalence of CVD and its related risk factors [8].

The elevated levels of the lipid profile (36% of the whole studied population) are the most prominent finding of the current epidemiological analyses, as they are raised in CVD patients (Table 1), smokers (Table 4) and subjects with raised BMI (Table 5) in the study population. Dyslipidemia is the principal cause of the excess burden of CAD in South Asians, which is characterized by increased levels of apolipoprotein (apo) B, TG, Lipoprotein Lp(a) and LDL-C, and low levels HDL-C and apoA1 [9]. The liberal use of saturated and trans fats in daily cooking especially curry-based cuisines and extensive deep frying along with lack of physical activity are one the profound features of Pakistani culture, thus leading to raised lipid levels. Previous studies in our population have provided diverse results; Jafar et al. reported high TC levels in 34.5% [10], Kayani et al. documented 10% [11] and Khan et al. found 16% of all study subjects had raised cholesterol levels [12]. In 2011, a study in Lancet reported the mean TC levels in the Asians not the highest but they were continuously on the rise owing to the epidemiological transition towards urbanization [13]. The increased consumption of carbohydrates owing to inflation making fresh fruits and organic food beyond the reach of a common man can also be one of the reasons for unhealthy dietary practices. Another study documented a consumption of 51.5% carbohydrates and 36.3% fats among urban population of Pakistan. There is an urgent need to inculcate the awareness of the less expensive and healthier food choices among people. They should be educated and encouraged towards adapting a better lifestyle within the means of their socioeconomic bracket. Therefore, careful and in-depth studies are required to develop these strategies and their implementation requires public awareness campaigns and government support.

The variations in the prevalence of CVD with respect to gender have also been investigated in the current study. In the whole studied population females had increased lipid levels as compared to males (Table 2). Furthermore, this increased vulnerability became more prominent when the patients were analyzed according to gender (Table 3). Previously, a high propensity of CVD in Pakistani women has been reported. Jafar et al. initially reported that women are at equally high risk of developing CVD as men [10]. Later on, the NHSP survey declared that women are at a greater risk for CVD than men [14]. Additionally, in 2008 more electrocardiographic evidence of ischemia was documented in women than in men [15]. In all these studies, increasing age has been hypothesized as the cause of increased prevalence of CVDs in women. However, in the current study the risk factors are more prominent at an early age (Table 6). The INTERHEART study (conducted in 52 countries) showed that males developed the myocardial ischemia eight years earlier than female counterparts; however, the infarction was more fatal in women [16]. Women in Pakistani culture are under educated, married at an early age, have no awareness about modifiable risk factors. The economic and psychological dependence on males restricts their access to health care. Another reason can be under recognition of CVD in women, different treatment strategies and decreased female representation in clinical trials. These facts emphasize on the urgent need to explore the reasons behind this enormous burden of CVD and the presence of risk factors in women.

Dyslipidemias along with other cardiovascular risk factors have been highly prevalent with increased BMI in South Indian adults [17]. Similar results were observed in the present study where the study population with increased BMI had raised SBP, DBP, TC, LDL-C and TC: HDL-C, clearly suggesting a significant impact of BMI on the CV risk factors. Similar results were observed by a study in Karachi documenting high prevalence of obesity [18]. Raised BMI is among one of the modifiable risk determinants and can easily be improved by indulging in physical activity and altering the dietary habits. This can be achieved through awareness campaigns and proper guidance by the health care providers and the community at large.

Advancing age has been associated with the increased prevalence of CVD in many countries [19]. In the United States, approximately 40% deaths in the population aged ≥65 years are due to atherosclerosis, stroke, heart failure and hypertension [20]. Although the formation of plaque is partly influenced by the chronological age of the individual, but the cardiovascular age is difficult to determine. The primary reason behind this is the influence of various CVD risk factors on the plaque formation at different points of life. The clinical and biochemical characteristics of the study population according to age were also analyzed (Table 6). Deranged lipid profile was significantly documented at a younger age as compared to old age. South Asians acquire the CVD risk approximately 10 years earlier compared to Caucasians and Chinese [21]. CVD-related mortality in people ≤70 years of age was 50% compared to 23% in western population [22]. Decreased prevalence of CVD risk was seen in adolescents but rapid escalation in these determinants was noted by age 30-39 years in urban Asian Indians [23]. The patho-physiological mechanisms are enhanced as the age advances leading to a pronounced effects on cardiovascular system in older individuals [19]. The present findings point towards increased vulnerability of Pakistani population towards CVD. It is also evident that because of this increased susceptibility, those individuals who are not having any disease at present are likely to develop CVDs later if not sooner.

## Conclusions

In conclusion, our study demonstrates the increased propensity of CVD in our population with female preponderance. Hypertension and dyslipidemia are the most prominent of the risk factors. Further research is required to determine the cause of this high burden of CVD in women so that appropriate measures can be undertaken to prevent significant morbidity and mortality. There is also a need to investigate the dietary habits and lifestyle of our population. In Pakistan, communicable diseases dominate the health care investment and the non-communicable diseases (NCDs) are ignored. This unfortunate trend needs to be changed because NCDs are now appearing as an “epidemic” which, if not dealt timely, will lead to catastrophe that will ruin our generations to come. The economic consequence of NCDs with increased incidence at a younger age along with lack of tertiary care will be huge, and thus unaffordable. This will place us in a vicious cycle which would be impossible to break. Our only option is to prevent it. Therefore, we need to develop a national awareness project to address this problem. It should be stepwise, starting from policy making at the government level, then awareness at the level of health care individuals and then awareness at the public level and lastly implementation of the policies. If we are able to target the risk factors earlier, then effective preventive interventions are there to reduce the prevalence of these diseases.
